# Calcium Homeostasis and Cone Signaling Are Regulated by Interactions between Calcium Stores and Plasma Membrane Ion Channels

**DOI:** 10.1371/journal.pone.0006723

**Published:** 2009-08-21

**Authors:** Tamas Szikra, Peter Barabas, Theodore M. Bartoletti, Wei Huang, Abram Akopian, Wallace B. Thoreson, David Krizaj

**Affiliations:** 1 Department of Ophthalmology, University of California San Francisco (UCSF) School of Medicine, San Francisco, California, United States of America; 2 Department of Ophthalmology & Visual Sciences, Moran Eye Center, University of Utah School of Medicine, Salt Lake City, Utah, United States of America; 3 Department of Ophthalmology & Visual Sciences, University of Utah School of Medicine, Salt Lake City, Utah, United States of America; 4 Department of Ophthalmology, New York University Medical Center, New York, New York, United States of America; 5 Pharmacology & Experimental Neurosciences Univ. of Nebraska Medical Center, Omaha, Nebraska, United States of America; 6 Department of Physiology, University of Utah School of Medicine, Salt Lake City, Utah, United States of America; Oregon Health & Science University, United States of America

## Abstract

Calcium is a messenger ion that controls all aspects of cone photoreceptor function, including synaptic release. The dynamic range of the cone output extends beyond the activation threshold for voltage-operated calcium entry, suggesting another calcium influx mechanism operates in cones hyperpolarized by light. We have used optical imaging and whole-cell voltage clamp to measure the contribution of store-operated Ca^2+^ entry (SOCE) to Ca^2+^ homeostasis and its role in regulation of neurotransmission at cone synapses. Mn^2+^ quenching of Fura-2 revealed sustained divalent cation entry in hyperpolarized cones. Ca^2+^ influx into cone inner segments was potentiated by hyperpolarization, facilitated by depletion of intracellular Ca^2+^ stores, unaffected by pharmacological manipulation of voltage-operated or cyclic nucleotide-gated Ca^2+^ channels and suppressed by lanthanides, 2-APB, MRS 1845 and SKF 96365. However, cation influx through store-operated channels crossed the threshold for activation of voltage-operated Ca^2+^ entry in a subset of cones, indicating that the operating range of inner segment signals is set by interactions between store- and voltage-operated Ca^2+^ channels. Exposure to MRS 1845 resulted in ∼40% reduction of light-evoked postsynaptic currents in photopic horizontal cells without affecting the light responses or voltage-operated Ca^2+^ currents in simultaneously recorded cones. The spatial pattern of store-operated calcium entry in cones matched immunolocalization of the store-operated sensor STIM1. These findings show that store-operated channels regulate spatial and temporal properties of Ca^2+^ homeostasis in vertebrate cones and demonstrate their role in generation of sustained excitatory signals across the first retinal synapse.

## Introduction

Daytime visual perception in diurnal animals is constrained by the sensitivity and operating range of retinal cones. Light-evoked cone signals are regulated by two separate gain control mechanisms: the phototransduction pathway at the input in the outer segment (OS) and the release rate of synaptic vesicles at the output in the synaptic terminal. Both pathways are dynamically regulated by changes in intracellular calcium concentration [Ca^2+^]_i_, which occur in the form of push-pull interactions between Ca^2+^ entry and clearance [reviewed in 1], [2]. Transmission of photopic stimuli is mediated through light-evoked [Ca^2+^]_i_ decreases in cone inner segments (IS) and synaptic terminals [3], which cause a decrease in exocytosis and activation of postsynaptic ON and OFF channels [4], [5]. Ca^2+^ homeostasis at the cone output may involve contributions by cGMP-dependent Ca^2+^-permeable channels (CNG channels) and intracellular Ca^2+^ stores [5]–[7]. However, the role of CNG channels and Ca^2+^ stores in generating transient and sustained signals downstream from the cone OS is unclear.

Ca^2+^ influx through L-type voltage gated channels stimulates neurotransmitter release in cones [3], [5]. However, cone synapses continue to transmit at hyperpolarized membrane potentials closer to −70 mV [8]–[10], although closure of cone L-type channels appears to be complete as cells hyperpolarize beyond ∼−50 mV [5], [11]–[13]. An inward rectifying current controlled by [cGMP]_i_ was suggested to extend the operating range of the cone output into the direction that is hyperpolarized vis à vis the L-type channel threshold [5], [14]. However, cone [cGMP]i is likely to decrease in saturating light, reducing the usefulness of this pathway for extending the operating range of cone neurotransmission.

We report a novel pathway in cone inner segments that dominates steady-state [Ca^2+^]_i_ baseline in hyperpolarized cones, potentially offsetting toxic effects of powerful Ca^2+^ clearance mechanisms [e.g., 15]. Activation of these Ca^2+^ - permeable channels is facilitated by hyperpolarization, potentiated by depletion of intracellular stores and is characterized by pharmacology that shares many features with store-operated Ca^2+^ entry (SOCE) that has been extensively studied in non-excitable cells [16]–[18]. Although Orai1 and TRPC channels that mediate SOCE in heterologously expressing systems are widely distributed throughout the brain [19], [20], there are only a few known physiological functions for SOCE in excitable cells [17], [21]–[24]. Our data using voltage-clamp and high-resolution optical measurements in single cells and retinal slices from the salamander retina suggests that these new channels provide a substantial contribution to sustained excitatory signaling in the cone pathway.

## Results

### Baseline of isolated cone photoreceptors is modulated by the driving force for Ca entry

Intracellular Ca^2+^ concentration in isolated salamander cones was measured by analyzing [Ca^2+^]_i_ signals from cells loaded with the high affinity indicator dye Fura-2. This non-invasive approach ensured that important cytosolic molecules potentially involved in modulation of Ca^2+^ fluxes were not lost or compromised. Salamander retina is advantageous for imaging studies because of the large size of salamander cone inner segments and synaptic terminals (5–10 µm diameter) [5], [6], [25]. Ratiometric dyes allow precise *in situ* calibration of cytosolic [Ca^2+^]_i_ within all classes of salamander cone [26]–[28].

**Figure 1:**
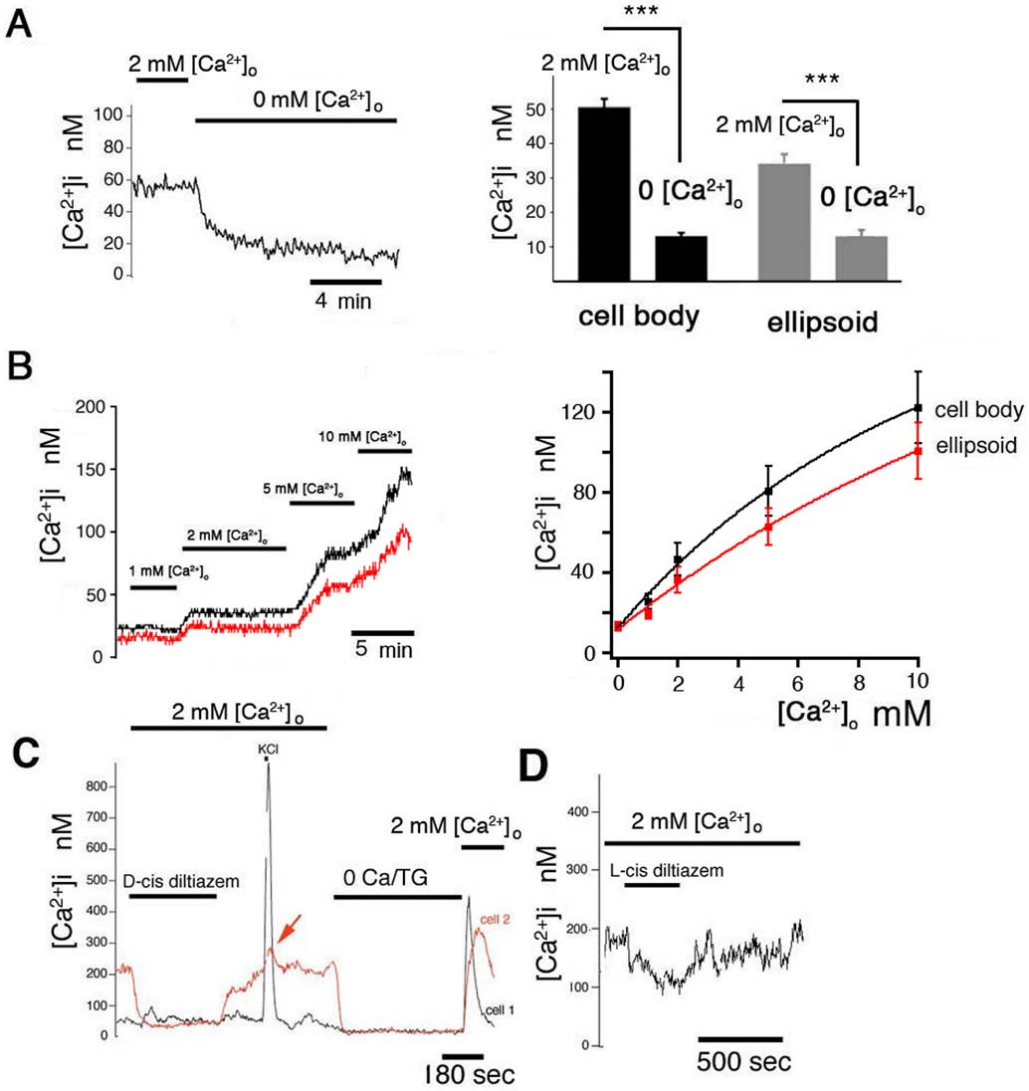
Ca^2+^ influx in the light adapted cone inner segment. (A) Removal of extracellular Ca^2+^ causes a sustained decrease in [Ca^2+^]_i_ in the cone perikaryon and ellipsoid. (B) *Left panel*: Cytosolic [Ca^2+^] in the cell body (black trace) and the ellipsoid (red trace) is a quasi-linear function of the driving force for Ca^2+^ entry. Ca^2+^ concentration in extracellular saline was modulated between 0 and 10 mM. *Right panel*: Cumulative data combining [Ca^2+^]_i_ responses to changes in [Ca^2+^]_o_. Data was fit with an exponential function. (C) Simultaneous recording from the two members of a double cone exposed to 200 µM D-cis diltiazem and 20 mM KCl. Cone 1 (principal member; black trace) was unaffected by diltiazem and responded strongly to depolarization. Following depletion of Ca^2+^ stores in 0 Ca^2+^/TG, the cell exhibited a fast monophasic increase in [Ca^2+^]_i_. Cone 2 (accessory member; red trace) had a high initial baseline [Ca^2+^]i which was lowered by diltiazem to ∼50 nM. Its response to depolarization was minimal (arrow). Depletion of internal stores triggered a monophasic overshoot in [Ca^2+^]_i_ in both cells. (D) L-cis diltiazem (10 µM) elicited a sustained decrease in [Ca^2+^]_i_.

Dissociated salamander cones consist of an ellipsoid attached to the cell body and synaptic terminal, but typically lack the small labile OS, which is lost during enzymatic and mechanical isolation procedures [27]. In the absence of the photocurrent, cones hyperpolarize below the threshold for activation of L-type Ca^2+^ channels therefore [Ca^2+^]_i_ tends towards a “resting” value associated with the saturating response to light. In dissociated light-adapted cones, the average resting [Ca^2+^]_i_ value in the cell bodies was low, at 50±3 nM. Removal of Ca^2+^ from extracellular saline caused a further [Ca^2+^]_i_ decline to 13±1 nM (n = 27, P<0.0001) with time constants of decay that were fit by a single exponential of τ = 2.0±0.3 min (Fig. 1A). A similar effect of nominally Ca^2+^-free saline was observed in the ellipsoid region in which [Ca^2+^]_i_ decreased from 34 to 13 nM (n = 23; P<0.0001; Fig. 1A, right-hand panel). This data suggests that [Ca^2+^]_i_ in isolated cones is sustained by Ca^2+^ entry from the extracellular space. Because baseline [Ca^2+^]_i_ values at the cone output define the lower limit of the dynamic range of cone neurotransmission [3], we next investigated the mechanisms that control steady-state [Ca^2+^]_i_ in light-adapted cones.

If hyperpolarized cones experience sustained influx of Ca^2+^, [Ca^2+^]_i_ in the IS should be sensitive to modulation of the driving force for Ca^2+^ entry. Ten-fold elevation of [Ca^2+^]_o_ from 1 mM to 10 mM resulted in approximately five-fold [Ca^2+^]_i_ increase in the cell body, from 26±4 to 123±18 nM (n = 23; P<0.001). Perfusion with control 2 mM Ca^2+^-containing saline led to [Ca^2+^]_i_ levels similar to control values (47±8 nM; Fig. 1B, black trace). The increase in the Ca^2+^ driving force also elevated ellipsoid [Ca^2+^]i (from 19 to 101 nM, n = 22; P<0.001; Fig. 1B, red trace).

Baseline Ca^2+^ entry was strongly suppressed by nonspecific lanthanide inhibitors of plasma membrane Ca^2+^ channels. Micromolar concentrations (10 µM) of Gd^3+^ and La^3+^ reduced [Ca^2+^]i baseline to 24±4 nM (n = 10; P<0.001) and 24±1 nM (n = 6; P = 0.0016), respectively. This suggests that tonic Ca^2+^ entry observed in hyperpolarized cones occurs via Ca^2+^ channels located in the plasma membrane.

We next tested whether voltage-operated channels, which are believed to represent the main Ca^2+^ influx pathway in the cone IS [2], [5], [12] contribute to baseline [Ca^2+^]i. 0 [Ca^2+^]_o_-induced decline in [Ca^2+^]i was not mimicked by Cd^2+^, a universal inhibitor of voltage-operated Ca^2+^ channels. At 100 **µ**M, Cd^2+^ had no effect on [Ca^2+^]_i_ (n = 16). Consistent with this observation, two antagonists of L-type channels, D-cis-diltiazem (100–200 **µ**M; n = 12) and verapamil (50 **µ**M; n = 3), did not affect [Ca^2+^]_i_ when applied to cones with ‘low baseline’ [Ca^2+^]_i_ (<100 nM). This is exemplified in the double cone depicted in Fig. 1C, in which the principal member exhibited high [Ca^2+^]_i_ and sensitivity to diltiazem (cell 2; red trace) whereas the accessory member was unaffected by the drug (cell 1; black trace). The average baseline in cones that did not respond to D-cis diltiazem was 42±5 nM (n = 24), not statistically significant from the cohort of total cones. In contrast, the average baseline [Ca^2+^]_i_ in which D-cis stereoisomer decreased [Ca^2+^]_i_ was 95±21 nM (n = 14), significantly different from healthy controls (P<0.001). Finally, exposure to diltiazem decreased baseline [Ca^2+^]_i_ to 56±17 nM, not statistically different from healthy non-treated cones (Fig. S1), but not to levels observed in Ca^2+^-free saline. Depolarizations that evoked large [Ca^2+^]_i_ elevations in hyperpolarized cells, produced little voltage-evoked response in presumed depolarized cells (Fig. 1C, arrowhead in red trace), in all probability because L-type channels in these cells were already open or inactivated. This data shows Ca^2+^ influx in dissociated cones with elevated [Ca^2+^]_i_ is mediated by L-type channels. However, complete suppression of voltage-operated Ca^2+^ entry leaves a component of Ca^2+^ entry that is mediated by a different class of ion channels and appears to be functional in all cones.

CNG channels were proposed to mediate Ca^2+^ entry in salamander cones hyperpolarized below −40 mV [5], [14]. L-cis diltiazem, which at 10 µM antagonizes photoreceptor CNG channels with higher affinity than L-type channels [29], [30] had little effect on baseline [Ca^2+^]_i_ in 8/11 cells. A reversible [Ca^2+^]_i_ decrease was observed in 3 L-cis diltiazem –treated cones (Fig. 1D), suggesting that CNG channels could supply a fraction of steady-state [Ca^2+^]_IS_.

In addition to plasma membrane Ca^2+^permeable channels, Ca^2+^ signaling in cone inner segments is also affected by ryanodine stores localized to the ER [6]. As opposed to large ER store signals in rods, direct Ca^2+^ release with fluorescent indicator dyes has been challenging to measure in cone inner segments, due to powerful Ca^2+^ extrusion system apposed to ER release sites [6]. We therefore studied the contribution of cone ER to plasma membrane Ca^2+^ signals by following changes in cone inner segment [Ca^2+^]_i_ after experimental manipulation of ER store content and through direct measurement of depletion-sensitive ion currents.

### Store depletion facilitates Ca^2+^ influx into cones

**Figure 2:**
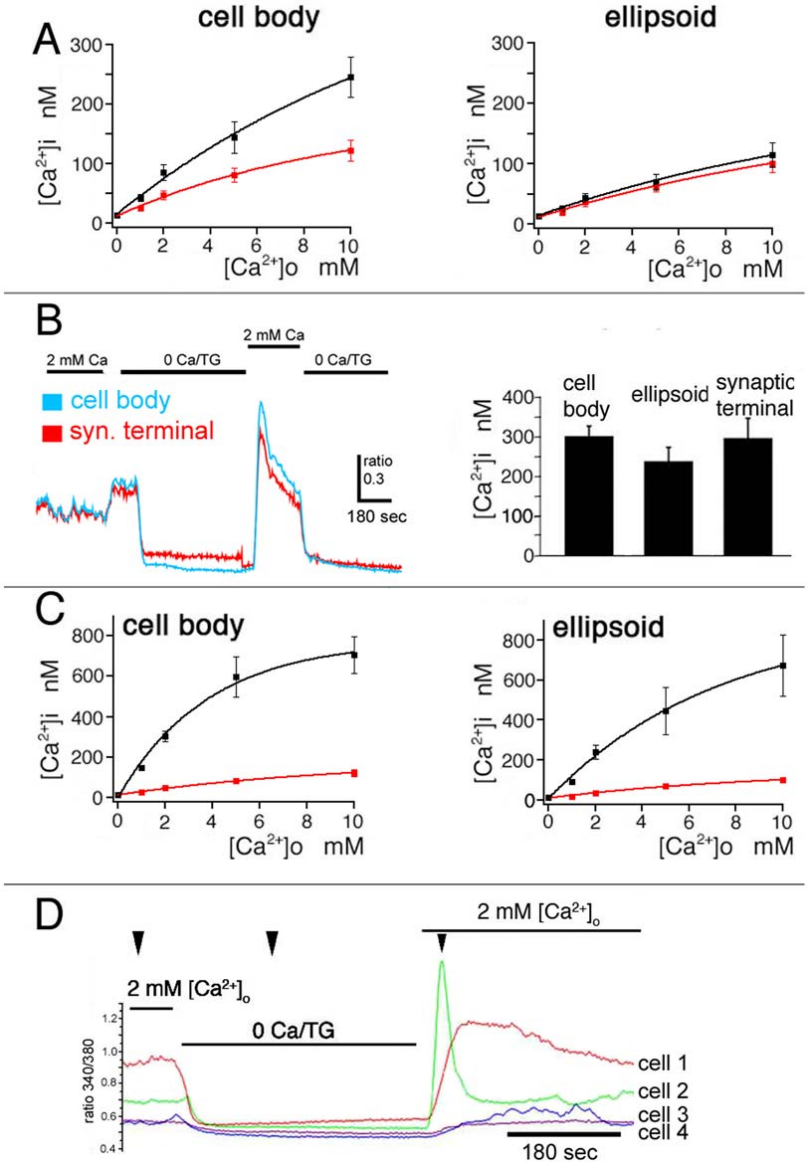
SOCE in cone photoreceptors. (A) Baseline [Ca^2+^]_i_ in cones is a function of [Ca^2+^]_o_; red trace represents cells with intact stores; black trace represents the same cells following exposure to 1 µM thapsigargin (TG). Thapsigargin treatment facilitates plasma membrane Ca^2+^ entry in the cell body (*left panel*) more than in the ellipsoid regions (*right panel*). (B) Ca^2+^ store depletion modulates Ca^2+^ entry in all compartments of the cone inner segment. *Left panel*: simultaneous [Ca^2+^]i recording from cell body (blue trace) and synaptic terminal (red trace) during store depletion in Ca^2+^-free saline and following re-addition of 2 mM Ca^2+^ reveals Ca^2+^ overshoots in both compartments. *Right panel*: Summary of Ca^2+^ overshoot data for synaptic terminal, cell body and ellipsoid [Ca^2+^]i elicited by the depletion protocol. (C) Superposition of [Ca^2+^]i baseline (in the presence of TG; red trace) and Ca^2+^ overshoots (following store depletion; black trace) for the cell body (*left panel*) and ellipsoid (*right panel*). (D) Four single cones under control conditions, during store depletion and during the Ca^2+^ overshoot phase (arrowheads). Cytosolic [Ca^2+^]_i_ in all four cells reaches the same level during store depletion but exhibit different amplitude and kinetics during the Ca^2+^ overshoot phase.

The effect of store depletion was ascertained directly by following exposure to the sesquiterpene sarcoplasmic-endoplasmic Ca^2+^ ATP-ase (SERCA) antagonist thapsigargin (TG; 1 µM). In 2 mM Ca^2+^-containing control saline, thapsigargin elevated [Ca^2+^]_i_ levels to 85±13 nM. In such cones, a potentiated sensitivity of cytosolic [Ca^2+^]_i_ for external Ca^2+^ entry was observed. Elevation of [Ca^2+^]_o_ from 1 mM to 10 mM resulted in an approximately six-fold [Ca^2+^]i change in TG-treated cells, elevating cytosolic [Ca^2+^] from 42±6 nM to 245±33 nM (n = 9; P = 0.012; Fig. 2A left panel). Exposure to thapsigargin had no significant effect on baseline [Ca^2+^]_i_ within the ellipsoid region (Fig. 2A right panel). These findings suggests that irreversible blockade of SERCAs facilitates activation of a plasma membrane Ca^2+^ influx pathway. We next investigated whether cone inner segments express store-operated Ca^2+^-permeable channels that work in concert with SERCA transporters to maintain steady-state cytosolic [Ca^2+^]_i_.

**Figure 3:**
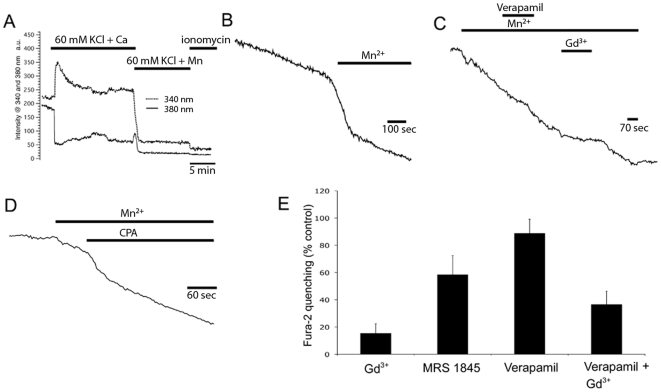
Tonic Mn^2+^ influx into ‘resting’ cones is modulated by calcium stores. (A) Cone depolarized with 60 mM KCl. 100 µM Mn^2+^ quenches Fura-2 fluorescence evoked by 340 and 380 nm excitation. Addition of 10 µM ionomycin evokes little additional quenching, indicating that most of the indicator dye is confined to the cytosol. (B) Fura-2 fluorescence evoked by 360 nm excitation. At rest, the dye bleaches at a rate that reflects loading and excitation strength (arrowhead). Addition of 50 µM Mn^2+^ causes quenching of the dye; (C) The rate of quenching is insensitive to verapamil (50 µM) and antagonized by Gd^3+^ (10 µM). (C) CPA (5 µM), increased the slope of Fura-2 quenching in a subset of cones. (D) Summary of the data for Mn^2+^ quenching experiments.

**Figure 4:**
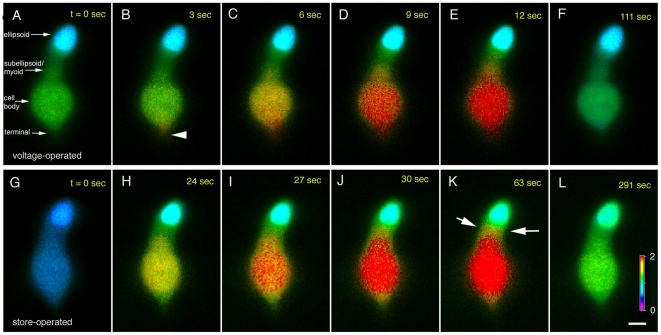
The spatial pattern of cone [Ca^2+^]_i_ during depolarization and SOCE. (A) Control. (B) 3 sec after stimulation with 20 mM KCl, [Ca^2+^]i starts to increase in the synaptic terminal. (C–E) [Ca^2+^]_i_ during continued exposure to 20 mM KCl is elevated in the cell body and terminal; modest increases take place in the subellipsoid and ellipsoid regions. (F) Washout. (G) [Ca^2+^]_i_ during store depletion in 0 Ca^2+^/TG saline. (H–K) SOCE during the Ca^2+^ overshoot. Large [Ca^2+^]_i_ elevation is observed in the perikaryon, followed by the subellipsoid and synaptic terminal regions. The time interval following beginning of depolarization (upper panels) and SOCE (lower panels) is shown at the upper right corner. Scale bar = 5 µm; ratio bar from 0.0–2.0.

Store-operated channels, which represent a main Ca^2+^ entry pathway in non-excitable cell [16], have been increasingly identified as contributors to neuronal Ca^2+^ homeostasis [31]–[34]. Typically, SOCE is identified by depleting ER stores in Ca^2+^-free solutions followed by re-exposure of cells to mM Ca^2+^-containing saline [e.g.], [ 34–36]. This protocol reliably evoked Ca^2+^ elevations in all cone inner segment compartments (Figs. 2B, 3 and 4). “Ca^2+^overshoots” observed in store-depleted cones were monophasic in the majority of cells, exhibiting a gradual [Ca^2+^]_i_ decline to a sustained plateau which remained elevated over the initial baseline [Ca^2+^]_i_ (Figs. 2B, D & 4A, B). However, the kinetics and amplitude of the overshoot varied in subsets of cones. Fig. 2D depicts an extreme example of four simultaneously recorded cones that were exposed to store depletion in Ca^2+^- free saline supplemented with TG. The four cells were classified as small single cones following Mariani's nomenclature [25]. Although [Ca^2+^]_i_ reached similar minima in all cells, cone 1 responded to store depletion with a sustained [Ca^2+^]_i_ plateau; cone 2 exhibited a large monophasic transient increase whereas cones 3 and 4 responded with a small delayed [Ca^2+^]_i_ increase and no change, respectively. Thus, cone inner segments can exhibit complex amplitude and temporal [Ca^2+^]_i_ patterns following depletion of ER stores and activation of SOCE.

The amplitude of depletion-evoked [Ca^2+^]_i_ overshoots was similar in the cell body and synaptic terminal (305±25 nM; n = 35, and 300±50 nM; n = 3) while lesser [Ca^2+^]i increases were observed in the ellipsoid region (241±35 nM (n = 12) (Fig. 2B). The sensitivity of depletion-evoked [Ca^2+^]_i_ overshoots for changes in the Ca^2+^ driving force was similar to the sensitivity of [Ca^2+^]_i_ baseline to changes in [Ca^2+^]_o_ with half maxima at 2.36 mM and 3.26 mM [Ca^2+^]_o_, respectively (Fig. 2C).

It is likely that most of the Ca^2+^ entry that occurs in resting cones is masked by powerful extrusion systems mediated by the PMCA transporters [6], [26]. To directly visualize the tonic activation of Ca^2+^-permeable channels in hyperpolarized cones we employed the Mn^2+^ quenching protocol in cones loaded with the Fura-2 indicator dye. Mn^2+^ ions appear to traverse most Ca^2+^-permeable channels; upon entering the cell, Mn^2+^ irreversibly binds Fura-2 and quenches its fluorescence [37], [38]. This is illustrated in Fig. 3A for Mn^2+^ influx through L-type voltage-operated channels activated by depolarizing the cone with high KCl. Depolarization increased the Fura-2 emission ratio, exemplified by the opposite response to the 340/380 nm excitation wavelengths. Fluorescence elicited by both wavelengths was quenched by 100 µM Mn^2+^ whereas 10 µM ionomycin produced little additional quenching, indicating that most of the Fura-2 is confined to the cytosol of the cone inner segment. At the [Ca^2+^]-independent isosbestic excitation wavelength (360 nm), exposure to Mn^2+^ elicited quenching of Fura-2 fluorescence (Fig. 3B); this effect was observed in all cones studied (n>60), demonstrating that ‘resting’ cones experience sustained influx of divalent cations. The data corrected for bleaching of the dye is shown in Fig. 3E. The L-type channel antagonists verapamil (50 µM; n = 10) (Fig. 3C & E) or nifedipine (n = 5; data not shown) had no effect on the slope of Fura-2 quenching whereas marked suppression of the quenching rate was observed following exposure to gadolinium (10 µM; n = 24) (Fig. 3B, C & E). MRS 1845, an antagonist of SOCE, reduced the influx by 55±9% (n = 11) whereas cyclopiazonic acid (CPA; 5–10 µM), an antagonist of SERCA transporters, increased the quenching rate in 5/25 cones. An example of a cell in which CPA increased the rate of quenching is shown in Fig. 3D. These results substantiate the observation that ‘resting’ cones experience tonic divalent influx that is sensitive to the loading state of internal Ca^2+^ stores.

Cone SOCE is spatially represented in Figure 4. The IS of the large single cone was stimulated with 20 mM KCl in order to activate voltage-operated Ca^2+^ entry (panels A–F; Video S1). As expected, this protocol triggered rapid [Ca^2+^]_i_ elevation in the synaptic region (arrowhead in B) from which Ca^2+^ spread into the cell body and the myoid/subellipsoid. Cytosolic [Ca^2+^] within the ellipsoid remained low, possibly due to mitochondrial uptake [28]. The spatial and temporal properties of SOCE displayed in panels G–L exhibited a number of differences compared to voltage-operated signals (Video S2). In contrast to rapid depolarization-evoked Ca^2+^ signals, the kinetics of the [Ca^2+^]_i_ increase after induction of SOCE were slow (panel H). Second, a quasi-uniform [Ca^2+^]_i_ increase was earliest observed across the nuclear area which is replete with ER cisternae [39]. Finally, Ca^2+^ elevation induced by SOCE diffused into the subellipsoid space (arrows in K) as a result of blocked SERCA-mediated Ca^2+^ sequestration, underscoring the role of the ER in regulation of subellipsoid and myoid [Ca^2+^]_i_. This data demonstrates that SOCE exhibits a distinct spatiotemporal signature that can be distinguished from previously reported modes of Ca^2+^ entry in cone photoreceptors.

**Figure 5:**
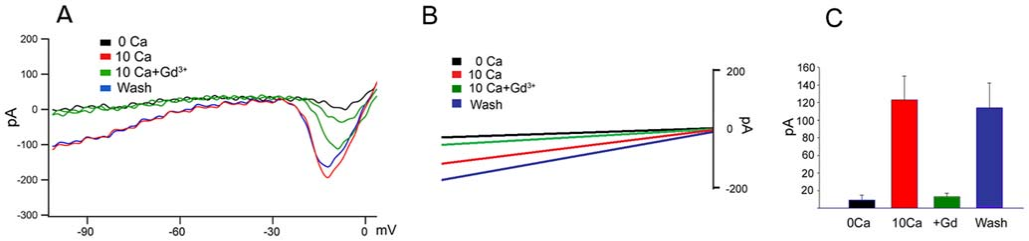
Voltage clamp in isolated cones in the retinal slice. (A) 250 msec voltage ramps from −150 to 0 mV in the presence of 5 mM external and internal CsCl and 10 mM [Ca^2+^]o elicited a transmembrane current that disappeared in the absence of Ca^2+^. The current shows inward rectification and is antagonized by 5 µM Gd^3+^. (B) Same experiment in the presence of 100 µM Cd^2+^. (C) Summary of data for 4 cones.

Although SOC currents are typically very small [16], we hypothesized that the current mediating Ca^2+^ influx in ‘resting’ cones might be maximized by combination of hyperpolarization and increased [Ca^2+^]_o_. Cone inner segments in the salamander retinal slice were voltage-clamped and stimulated with voltage ramps ranging from −150 to 0 mV in the presence of 10 mM external Ca^2+^. After the I_h_ cation conductance was blocked with internal and external Cs^+^, little transmembrane current was observed in control saline. However, depletion of ER stores with thapsigargin combined with an increase in transmembrane Ca^2+^ gradient evoked significant currents (123±27 pA; n = 5) in the presence of Cs^+^. The hyperpolarization-evoked current was blocked by elimination of extracellular Ca^2+^ (9±3 pA, n = 5) or Gd^3+^ (5 µM) (12±4 pA, n = 3) (Fig. 5). The Ca^2+^ current evoked by hyperpolarization persisted in the presence of 100 µM Cd^2+^ (Fig. 5B). This data shows that cone hyperpolarization activates calcium influx that is antagonized by SOCE blockers.

### Store depletion-evoked [Ca^2+^]_i_ changes are suppressed by SOCE antagonists

**Figure 6:**
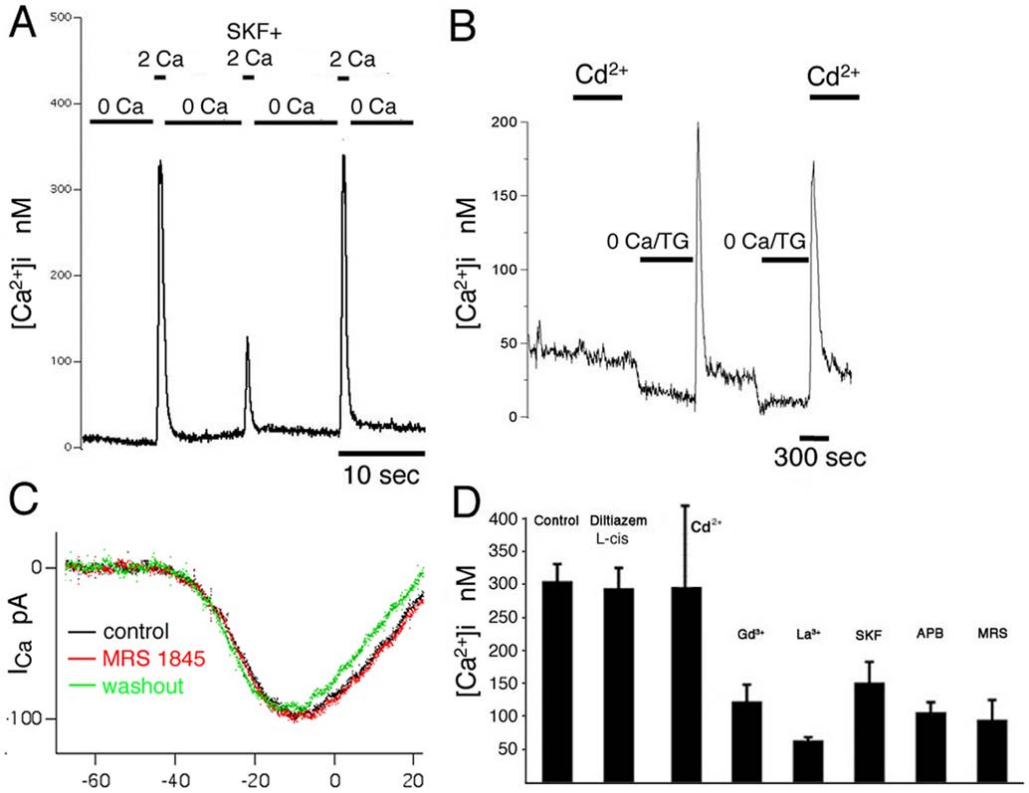
Depletion-evoked Ca^2+^ overshoots are suppressed by SOCE antagonists and are unaffected by L-type and CNG channel antagonists. (A) Ca^2+^ overshoots are reversibly suppressed by 5 µM SKF 96365. (B) 100 µM Cd^2+^ has no effect on baseline [Ca^2+^]_i_ or depletion-evoked Ca^2+^ overshoots. (C) Voltage-clamped cone stimulated by a depolarizing ramp (−90 to +50 mV, 0.5 mV/ms) before (black trace), during (red trace) and after superfusion with 25 µM MRS 1845. (D) Summary of data for Ca^2+^ overshoots in cones treated with L-cis diltiazem (10 µM), Cd^2+^ (100 µM), Gd^3+^ (10 µM), La^3+^ (10 µM), SKF 96365 (5 µM), 2-APB (100–200 µM) and MRS 1845 (15 µM).

Inhibition by low (µM) concentrations of lanthanides and organic antagonists such as SKF 96365 or 2-APB is considered to be diagnostic for SOC channels [16], [36]. The most commonly used inhibitor of SOCE is the econazole SKF 96365 (1-[β-[3-(4-methoxyphenyl)propoxy]-4-methoxyphenethyl]-1H-imidazole). 5 µM SKF 96365 significantly reduced the amplitude of depletion-induced Ca^2+^ overshoots in cones (to 151±31 nM; n = 3) (Fig. 6A). The mean change in [Ca^2+^]_i_ after exposure to SKF 96365 was ∼50% suppression, significantly different from cells treated with thapsigargin alone (P = 0.032). 2-APB (2-aminoethoxy-diphenylborate) (100–200 µM), another typical inhibitor of SOC channels, suppressed depletion-evoked Ca^2+^ signals by ∼70% (to 135±19 nM; n = 6; P = 0.047; Fig. 6D). Likewise, 10 µM Gd^3+^ reduced the amplitude of the Ca^2+^ overshoot by ∼60% (to 123±25 nM; n = 9; P = 0.025). Application of another lanthanide, La^3+^ (10 µM), resulted in ∼80% inhibition (to 64±5 nM; n = 7; P = 0.027) (Fig. 6D) of the overshoot.

MRS 1845, a recently developed dihydropyridine promoted as a high-affinity SOC channel antagonist [40] also reduced the amplitude of SOCE (the overshoot in the presence of 15 µM MRS 1845 was 95±30 nM; n = 4; P = 0.041, Fig. 6D). Unlike the lanthanide and organic inhibitors of SOCE, MRS 1845 does not inhibit voltage-operated Ca^2+^ channels: the drug had no effect on the amplitude or the time course of voltage-operated Ca^2+^ currents (Fig. 6C) and/or depolarization-evoked [Ca^2+^]_i_ elevations in salamander cones (see below), rods [34] and ganglion cells (data not shown). In contrast to the efficacy of SOC antagonists, high concentrations of Cd^2+^, the universal voltage-activated Ca^2+^ channel antagonist, had no effect on the amplitude of Ca^2+^ overshoots. In 15 cells tested, peak SOCE was 237±83 nM, not significantly different from 299±122 nM in 100 µM Cd^2+^ (P = 0.585; Fig. 6D).

We next tested the effect of the CNG channel antagonist L-cis-diltiazem on SOCE. In the presence of 10 µM diltiazem, the amplitude of [Ca^2+^]_i_ overshoots averaged 294±30 nM (n = 5), not significantly different from control values (data not shown). This suggests that depletion of ER stores does not modulate CNG-mediated Ca^2+^ signaling in cone ISs. Taken together, this data suggests that cone photoreceptors express a plasma membrane Ca^2+^-permeable pathway that exhibit a pharmacological profile characteristic of SOC channels.

### Is SOCE activated in depolarized cones?

The results presented above suggest that SOCE operates in hyperpolarized light-adapted cones. We next investigated whether SOCE can contribute to signaling in depolarized cones. Presumed depolarized cones were identified by elevated baseline [Ca^2+^]_i_ (>50 nM), sensitivity of [Ca^2+^]_i_ baseline to D-cis-diltiazem and insensitivity to further depolarization (e.g.; arrow in Fig. 1C). Depletion of ER stores in 0 Ca^2+^/TG saline decreased [Ca^2+^]_i_ in depolarized cones to the level observed in hyperpolarized cones. Nonetheless, as shown in Fig. 1C, [Ca^2+^]_i_ overshoots in depolarized cells had comparable amplitudes to overshoots measured in hyperpolarized cells, suggesting that SOCE can be activated across the operating range of cone function.

**Figure 7:**
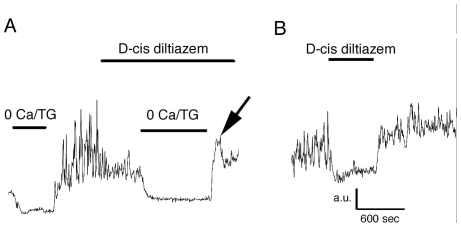
Induction of SOCE was associated with [Ca^2+^]_i_ plateau that was suppressed by 200 µM D-cis diltiazem. The drug did not block the Ca^2+^ overshoot (arrow). (B) Same cell during the sustained plateau following store depletion. The elevated [Ca^2+^]_i_ baseline is suppressed by diltiazem. Uncalibrated cone; a.u. = arbitrary units.

The amplitudes of Ca^2+^ overshoot and/or [Ca^2+^]_i_ plateaus following induction of SOCE were not invariant with respect to contribution of Ca^2+^ influx via L-type channels in all cones. As observed above in cones with elevated [Ca^2+^]_i_ baselines, depletion-induced Ca^2+^ overshoots and plateaus were attenuated in a subset of cells by D-cis diltiazem (200 µM) and verapamil (50 µM). Occasionally, depletion-evoked overshoots continued as sustained [Ca^2+^]_i_ plateaus that were partially sensitive to L-type channel antagonists (Fig. 7). Table 1 tabulates data obtained from cones exposed to D-cis diltiazem. D-cis diltiazem had no effect on the Ca^2+^ overshoot in 46% of cells (n = 17/37), suggesting that depolarization evoked during the induction of SOCE was insufficient for crossing the threshold for L-type channel activation. The drug suppressed the amplitude of the overshoot more strongly in cones in which [Ca^2+^]_i_ baseline was already elevated; in contrast, Ca^2+^ overshoots in most (71%) of the cones that exhibited no change in baseline response to D-cis diltiazem, were also insensitive to the drug (Table 1). Similar results were obtained using the L-type blocker verapamil (n = 4; data not shown). These data suggest that cation entry through SOC channels can facilitate activation of voltage-operated Ca^2+^ influx, especially in slightly depolarized cones that are close to the L-type Ca^2+^ channel threshold.

**Table 1:** Store operated response in salamander cones shows mixed responses to L-type inhibition.

	SOCE Decrease	SOCE No change	SOCE Increase	SOCE Sum
Baseline Decrease	8	3	1	12
Baseline No change	9	12	2	23
Baseline Increase	0	2	0	2
Baseline Sum	17	17	3	37

Effect of 200 μM D-cis diltiazem on baseline [Ca^2+^]_i_ and SOCE in cone inner segments (n = 37). In most cells, neither baseline [Ca^2+^]_i_ nor SOCE were affected by D-cis diltiazem. A subset of cones responded to L-type inhibition with both baseline decreases (left- and upper-most cell). The baseline response is plotted in the left-most column. SOCE responses (measured as peak amplitudes of depletion-evoked Ca^2+^ overshoots) are plotted on top.

### SOCE suppression decreases cone-evoked signals in postsynaptic cells

**Figure 8:**
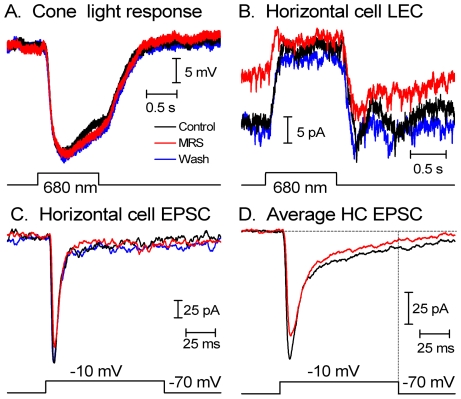
SOCE modulates cone neurotransmission. (A) The SOC channel antagonist MRS-1845 (15 µM) had no effect on cone light responses evoked by 1 sec 680 nm flashes. (B) The light-evoked current recorded from a horizontal cell in cone-dominated conditions (red light flash applied in the presence of a dim blue adapting background light) was significantly reduced by bath application of MRS-1845. Consistent with a reduction in tonic glutamate release, MRS-1845 also caused an outward shift in the horizontal cell membrane current prior to the light flash. (C) In a simultaneously recorded cone and horizontal cell pair, depolarizing the presynaptic cone to −10 mV for 100 ms evoked a PSC in the horizontal cell consisting of both fast transient and slower sustained components. MRS-1845 caused a small reversible decrease in the PSC. (D) Averaged data from 9 horizontal cells. The dotted vertical line at the end of the depolarizing step illustrates the difference in the averaged sustained slow component between control (black trace) and MRS 1845-treated (black trace) HCs. In all experiments illustrated in this figure, MRS-1845 was bath applied for 3–4 min.

**Figure 9:**
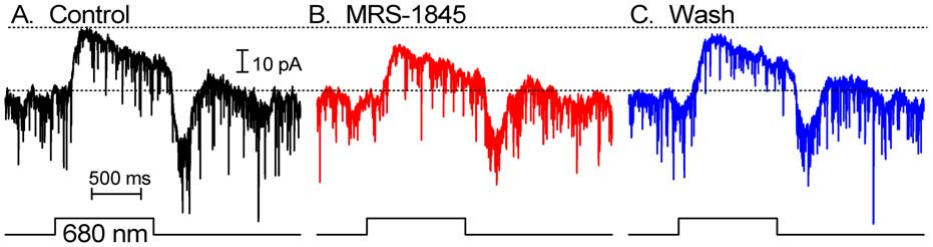
MRS 1845 suppresses OFF bipolar cell light responses. The light-evoked current recorded from an OFF bipolar cell in cone-dominated conditions (red light flash applied in the presence of a dim blue-adapting background light) was reversibly inhibited by bath application of MRS-1845 (15 µM). There was a steady inward baseline current drift during this recording. The dark current levels preceding OFF bipolar cell light responses in Fig. 9 were aligned (dotted horizontal lines) to better illustrate the reduction in LEC amplitude. The lower dashed line shows the membrane current in darkness under control conditions; the upper dashed line shows the level attained during the light-evoked current.

Localization of SOCE to the cone synaptic terminal (Figs. 2 and 4) suggests that store-operated channels could influence neurotransmission at cone synapses. We tested this hypothesis with the SOCE antagonist MRS 1845. MRS 1845 (15 µM) had no significant effect on cone light responses (+9.8 ±11.7%, N = 4, Fig. 8A), voltage-dependent I_Ca_ in cones (+1.3 ±6.5%, N = 7, Fig. 6C) or ganglion cells (n = 3; data not shown) nor did it affect depolarization-evoked [Ca^2+^]i increases in rods [34], suggesting this dihydropyridine does not block voltage-operated Ca^2+^ channels or ion fluxes that shape the cone light response. Surprisingly, 15 µM MRS 1845 caused a 48.3 ±10.6% reduction in the amplitude of cone-dominated light-evoked responses of horizontal cells (N = 7; P = 0.003; Fig. 8B). Consistent with a presynaptic site for this inhibitory effect, cone-dominated light-evoked responses of OFF bipolar cells were also inhibited by 15 µM MRS 1845 (−25.7+9.6%, P = 0.044, N = 6) (Fig. 9). As expected for a reduction in sustained release of glutamate from photoreceptors, MRS 1845 caused a significant outward shift in horizontal cell holding currents (+33.9±9.1 pA, P = 0.01, N = 7). The DC current in the bipolar cells shifted +3.0±3.1 pA (P = 0.37, N = 6) compared to the average DC current level before and after MRS1845. The small and statistically insignificant DC shift is not surprising given the small size of OFF bipolar cell light responses which averaged, under these highly light-adapted conditions, only 14.9±3.38 pA. The reduction in OFF bipolar cell light-evoked currents was not significantly less than the reduction in HC light-evoked currents (P = 0.1, unpaired t-test).

We next examined post-synaptic currents (PSCs) evoked in horizontal or OFF bipolar cells by strong depolarizing steps (−70 to −10 mV, 100 ms) applied to simultaneously recorded presynaptic cones. This stimulus evokes a fast inward current due to activation of AMPA receptors followed by a very small, sustained inward current [41]. Despite the prominent effect on the light response, MRS 1845 caused only a small reduction in the peak amplitude of post-synaptic currents evoked by strong depolarizing steps in the presynaptic cone (−11.4±3.6%, P = 0.01) in 9 cone/horizontal cell pairs (Fig. 8C) and 2 cone/OFF bipolar cell pairs (data not shown). Averaged data for 9 HCs, illustrated in Fig. 8D, show that suppression of SOCE has a disproportionately large effect on the slow sustained component of the evoked EPSC. At the end of the test step, the reduction in the sustained EPSC produced by MRS1845 approached ∼50% reduction, similar to the reduction in the amplitude of light-evoked HC currents (Fig. 8B) and the magnitude of MRS 1845-suppressed depletion-evoked [Ca^2+^]_i_ elevations in isolated cones (Fig. 6D). This data indicates that MRS 1845, and presumably SOCE, modulate sustained release from cones but have less effect on the initial burst of release associated with fast depletion of the presynaptic vesicle pool at the ribbon (Rabl et al., 2005, Cadetti et al., 2006).

In contrast to MRS 1845, nifedipine (30 µM), an antagonist of L-type Ca^2+^ channels, equally suppressed the transient and sustained components of the postsynaptic EPSC (nifedipine/control, peak = 0.73±0.05; sustained = 0.63±0.1; P = 0.17) (n = 4; Fig. S1A). L-cis diltiazem (10 µM) had no effect on the amplitude or kinetics of horizontal cell EPSCs (n = 5; Fig. S1B), suggesting CNGC channels do not contribute to the evoked EPSC at the cone synapse. To ascertain whether MRS 1845 modulates SOCE and postsynaptic excitatory signals in horizontal cells, the glutamate receptor agonist AMPA (100 µM) was applied onto horizontal cell dendrites with a puffer pipette in the presence and absence of MRS 1845. AMPA applications evoked similar current amplitudes in control (504 ±72 pA) and MRS 1845-containing saline (505 ±69; N = 4; P = 0.96), suggesting the drug's main effect on cone neurotransmission is presynaptic.

### STIM1, the central element of SOCE, is localized to salamander cones

**Figure 10:**
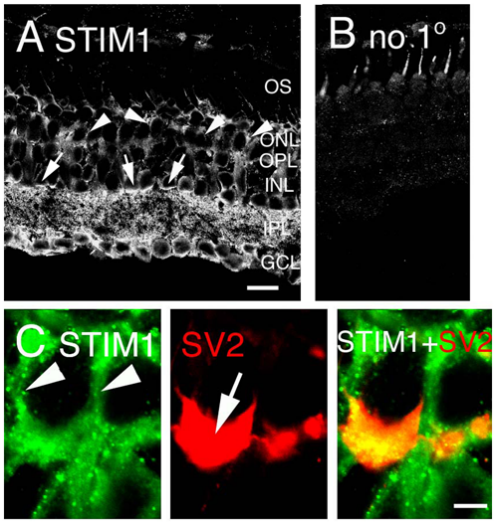
Immunolocalization of STIM1 in the salamander retina. (A) The rabbit polyclonal STIM1 antibody labeled most cells in the salamander retina, including cones (arrowheads). STIM1-immunoreactive signals were observed in presumed amacrine cells at the boundary of the IPL and INL (arrow). Bar = 20 µm. (B) Fluorescence signal in the absence of the primary antibody. (C) Double labeling for STIM1 and the synaptic vesicle marker SV2 shows modest colocalization in the cone pedicle (arrow in the middle panel). Scale bar = 2 µm. *Abbreviations*: OS, outer segment; ONL, outer nuclear layer; OPL, outer plexiform layer; INL, inner nuclear layer; IPL, inner plexiform layer; GCL, ganglion cell layer.

Recent RNAi screening studies showed that SOCE is activated by the clustering of the E-F hand protein STIM1 in the ER underneath the plasma membrane [42], [43]. STIM1 is believed to act as a sensor that communicates the Ca^2+^ loading state of the ER store to plasma membrane store-operated TRPC and/or Orai channels [44], [45]. Immunostaining of retinal sections with the polyclonal STIM1 antibody labeled most retinal neurons, including rod and cone photoreceptors. The cell bodies in the proximal ONL (corresponding to cones) were immunostained (Fig. 10A, arrowheads), however, the staining in rods was more prominent than in cones. Double labeling with the synaptic marker SV2 showed weak colocalization between STIM1 and crescent-shaped structures typical of salamander cone pedicles (Fig. 10C). Cell bodies, processes and endfeet of Müller glia were also labeled by STIM1 antibody, consistent with SOCE in these cells [46].

## Discussion

We investigated a Ca^2+^ influx mechanism that modulates the dynamic range of presynaptic Ca^2+^ signaling in vertebrate cones. While the mechanism operates maximally under light-adapted conditions it may also contribute to sustained signaling in the darkness. Unexpectedly, its activation is facilitated by depletion of ER Ca^2+^ stores, implicating SOCE in photopic signaling in the retina.

### Cone SOCE has a unique pharmacological and spatiotemporal signature

Restoring extracellular Ca^2+^ after depletion of Ca^2+^ stores in the cone inner segment transiently elevated [Ca^2+^]_i_ to several hundred nM and was typically followed by a prolonged [Ca^2+^]_i_ plateau. The pharmacological profile of this depletion-sensitive mechanism exhibited many hallmarks of SOCE, including sensitivity to the Ca^2+^ driving force and suppression by lanthanides and organic SOCE antagonists SKF-96365, 2-APB and MRS 1845. Irreversible inhibition of Ca^2+^ sequestration into the ER by thapsigargin both elevated baseline [Ca^2+^]i and potentiated the magnitude of Ca^2+^ transients generated by re-exposure to mM [Ca^2+^]_o_. Whereas micromolar concentrations of La^3+^ and Gd^3+^ decreased cytosolic baseline [Ca^2+^]_i_ close to the levels observed in [Ca^2+^]_o_-free conditions, in hyperpolarized cells this effect was not duplicated by L-cis-diltiazem, D-cis-diltiazem, verapamil or Cd^2+^, suggesting that baseline [Ca^2+^]_i_ transients are not maintained by either L-type nor CNG channels.

The half-maximal sensitivity for baseline Δ[Ca^2+^]i elicited by changes in the Ca^2+^ gradient was similar to concentration-dependence of Ca^2+^ “overshoots” observed in cells with depleted ER stores, suggesting that part of the standalone “resting” current in light-adapted cells derives from SOCE. Previous studies showed that a significant fraction of Ca^2+^ ions entering the cells through SOC channels is immediately pumped out of the cytosol by high-affinity PMCA transporters [47]. To determine whether SOC influx is masked by extrusion, we took advantage of an assay that bypassed activation of the PMCA pumps. Mn^2+^ can enter the cells through store-operated channels despite the fact that these channels are less permeable to Mn^2+^ than to Ca^2+^ [35]. Mn^2+^ quenching of the Ca^2+^ indicator dye revealed substantial sustained divalent cation influx into the hyperpolarized cone IS. This cation influx was highly reproducible, observed in all studied cones, was insensitive to antagonists of voltage-operated channels yet antagonized by the SOCE blockers Gd^3+^ and MRS 1845.

Ca^2+^ entry through store-operated channels is likely to parallel the proposed inwardly rectifying cation conductance mediated by CNG channels [5], [14]. However, in contrast to CNG channels which appear to be confined to synaptic terminals (Rieke and Schwartz, 1994) and L-type channels which, while predominantly localized to the terminals are also expressed in the soma [26]–[28], (Fig. 3I) the spatial pattern of depletion-evoked [Ca^2+^]_i_ signals in the cone inner segment emphasized the somatic compartment. While differing markedly from [Ca^2+^]_i_ gradients mediated by L-type channels in depolarized cones (Figure 3C), the distribution of SOCE matched localization of ER cisternae [39] and the ER-based SOC sensor STIM1. The time-course of depletion-dependent [Ca^2+^]_i_ changes in cone ISs was significantly slower compared to rapid [Ca^2+^]_i_ elevation evoked by depolarization or Ca^2+^ release from ryanodine stores. Hence, store depletion creates spatiotemporal Ca^2+^ signaling subcompartments in vertebrate cones that can be distinguished from signals mediated by L-type and CNG channels or ryanodine receptors [5], [6]. Notably, SOCE activation is associated with [Ca^2+^]_i_ homeostasis in the perikaryon (Fig. 4), suggesting that SOC channels could modulate transcription [e.g., 18]. Whilst cone [cGMP] is lowered by light, the likelihood of SOCE was maximized in light-adapted cells, i.e., under conditions when cone ER stores are depleted of Ca^2+^ [6]. We also note that the amplitude and kinetics of SOCE responses was much more variable than the conserved responses of voltage-operated Ca^2+^ channels, suggesting that SOCE is susceptible to modulation by intracellular signaling cascades. For example, the transient components of depletion-evoked overshoot could be modulated by Ca^2+^-calmodulin-mediated inactivation and phosphorylation [48] whereas PMCA pumps and mitochondria could regulate the steady-state component [e.g.], [47, 49].

### SOCE and cone survival

SOCE may protect cones by obviating potentially cytotoxic depletion of Ca^2+^ during prolonged light exposure. Considerable evidence suggests that photoreceptors and most neurons die when intracellular [Ca^2+^] becomes too high [50]–[52] or too low [15], [53] whereas modest elevation tends to be neuroprotective [54]. Continuous light triggers apoptotic photoreceptor cell death by over-activating the transduction cascade and lowering [Ca^2+^]_OS_ to toxically depleted levels [53]. Similarly, reduction of intracellular [Ca^2+^] triggers the apoptotic process in cerebellar granule cells [55]. We demonstrate a comparable phenomenon in the cone inner segment. Loss of tonic Ca^2+^ influx caused a rapid [Ca^2+^]_i_ decrease due to continued activation of PMCA transporters. If prolonged, depletion of Ca^2+^ lowers [Ca^2+^]_i_ near 0 nM and triggers apoptosis [TS, PB, DK, unpublished observations; 15] as sustained loss of ER Ca^2+^ results in impaired protein synthesis and protein folding [56]. However, it is possible that activation of SOCE through irreversible suppression of SERCA transporters eventually results in calcium overload and cell degeneration [57]. Our results suggest that the maximal membrane conductance associated with full activation of SOCE channels (combination of 10 mM external Ca^2+^ & hyperpolarization) is relatively low (Figure 5). Under physiological conditions, activation of SOCE following exposure to light stimuli that close the voltage-operated Ca^2+^ channels is likely to function as a robust neuroprotective mechanism with the primary aim to maintain translation, protein folding and ryanodine receptor function within the ER yet its contribution to overall Ca^2+^ flux in darkness would be relatively small.

### SOCE interacts with voltage-operated [Ca^2+^]i changes and modulates neurotransmission

We found that [Ca^2+^]_i_ levels higher than 100 nM are invariably associated with activation of L-type channels. A recent report using multiphoton microscopy found that [Ca^2+^]_i_ baseline in light-saturated *Anolis* lizard cones is relatively high after exposure to saturating white light (∼188 nM), limiting the dynamic range of presynaptic [Ca^2+^]_i_ to ∼two-fold [3]. It is possible that light-saturated anole cones contain a depolarizing component additional to the store-operated Ca^2+^ signal. Indeed, we found that steady-state [Ca^2+^]_i_ in partially depolarized salamander cones is determined through intimate interactions between SOCE and voltage-operated Ca^2+^ entry. This conclusion was suggested by pharmacological manipulations of Ca^2+^ overshoots, plateau [Ca^2+^]_i_ and Ca^2+^ oscillations by L-type and CNG channel antagonists in cells with depleted ER stores. However, in the majority of salamander cones, possibly corresponding to fully light-adapted cells, baseline [Ca^2+^]_i_ tended to range between 30 and 70 nM. These levels were unaffected by antagonists of voltage-operated Ca^2+^ entry and were almost entirely sustained by Ca^2+^-permeable channels that were sensitive to antagonists of many store-operated and TRP channels. Hence, the dynamic range of average presynaptic [Ca^2+^]_i_ in salamander cones appears to be at least ∼20–40 fold, ranging from ∼50 nM in the light to several µM measured in depolarized cone terminals [3], [27]. Local [Ca^2+^]i levels closer to the mouths of Ca^2+^ channels at the active zone are likely to be significantly higher in the tens of µM range [58].

Although SOCE itself is not activated by depolarization, Ca^2+^ influx through SOC channels contributes a depolarizing drive that brings the cone membrane potential closer to the threshold for activation of voltage-operated Ca^2+^ entry. Hence, SOCE collaborates with voltage-operated cation entry in the regulation of cone output, possibly by smoothening the transition between photopic and mesopic ambient conditions. Inhibition of SOCE might be predicted to suppress cone output signals both indirectly by reducing the tonic depolarizing drive and through direct action on SOC channels. Consistent with this hypothesis, the SOCE antagonist MRS 1845 partially reduced the amplitude of the horizontal and OFF bipolar cell light response while having less effect on the fast initial EPSC component (mediated by L-type channels). The reduction in OFF bipolar cell light-evoked currents was not significantly different from the reduction in horizontal cell light-evoked currents. Cell to cell differences in the effects of MRS1845 might arise from differences in the properties of AMPA receptors between horizontal cells, rod- and cone-driven bipolar cells and the possibility that some OFF bipolar cells may possess KA receptors [7], [41], [59]–[60]. If glutamate receptors of bipolar cells exhibit a higher affinity for glutamate than HCs, this could necessitate a greater decrease in glutamate release to produce the same reduction in LEC amplitude. In contrast to the effect of MRS 1845, inhibition of L-type channels had an equal effect on phasic and tonic components of evoked EPSCs (Fig. 8). Paired recordings from cone-horizontal cell pairs suggest that SOCE could modulate the slower and sustained components of the light response, possibly by regulating the exocytotic process as suggested in previous studies in excitable cells [21]–[23], [34]. Store depletion and SOCE in cone ISs could regulate, and be regulated by, a number of other signaling pathways, including ‘non-calcium’ pathways involving cAMP [61] and products of the lipid metabolism.

The molecular identity of channels that mediate the Gd^3+^-sensitive voltage-independent Ca^2+^ influx in cone photoreceptors is currently unknown. TRPC channels are among the major candidates for mediating SOCE [17], [34], [36], [44]. Previous studies have identified TRPC1, TRPC4 and TRPC5 channels in cortical synaptosome preparations [62], TRPC5 in growth cones [24], and TRPC1 in axonal processes of hippocampal CA1-CA3 pyramidal cells [19]. TRPC1, the only canonical TRP isoform so far cloned in amphibians [63], mediates ∼ 50% of SOCE in salamander rods but appears to be excluded from cones [34]. Amphibian TRPC1 homolog, activated by netrin-1, amplifies Ca^2+^ entry through voltage-operated Ca^2+^ channels, leading to the turning of growth cones [64]. Immunolocalization in macaque, mouse and rat retinas together with electrophysiological recordings from TRPC6-null mice [65] suggest that cones in several vertebrate retinas localize and functionally express TRPC6. Because TRPC6 channels are not always activated by store depletion [66], [but see 67], cone SOCE could be mediated by TRPC6 heteromers or other TRPC/Orai isoform combinations [45].

Taken together, our data suggests that SOCE integrates signaling pathways in the plasma membrane (SOC channels, L-type channels and CNG channels) with functional state of the intracellular Ca^2+^ store (and STIM1 sensors, SERCA transporters and ryanodine receptors) to produce a background Ca^2+^ signal which affects steady-state cone [Ca^2+^]_i_ as well as cone output signals. These signals may not only function in replenishment of ER stores, but also stimulate local Ca^2+^ pathways required for cone signaling and survival [68]. The SOCE mechanism is well placed to modulate the dynamic range of cone signaling beyond limits imposed by the activation range of voltage-operated Ca^2+^ entry.

## Materials and Methods

[Ca^2+^]_i_ concentration was measured in cone ISs loaded with the AM ester of the fluorescent indicator Fura 2 (fura 2-acetoxymethylester; Invitrogen, Eugene, OR) as reported previously [34]. Larval tiger salamanders (*Ambystoma tigrinum*) were decapitated and pithed using procedures recommended by the National Institute of Health Guide for the Care and Use of Laboratory Animals. Retinas were dissociated in 0 Ca^2+^/papain (10–30 U/ml; Worthington, Freehold, NJ) saline for 20 min at room temperature (20–22°C). Cells were plated onto coverslips coated with 0.2 mg/ml concanavalin A (Sigma, St. Louis, MO). The recording chamber was superfused via an electronically controlled multi-inlet manifold. The control saline solution contained, (in mM), 97 NaCl, 2 KCl, 2 CaCl_2_, 2 MgCl_2_, 10 HEPES, 2 lactic acid, 0.3 ascorbic acid and 1 taurine at 240 mOsm. pH was adjusted to 7.6 with NaOH. To stimulate glycolysis, glucose concentration in the saline was elevated to 20 mM. All salts and STIM1 antibody were obtained from Sigma. 2-APB, L- and D-cis diltiazem were from Biomol (Plymouth Meeting, PA); verapamil, thapsigargin and SKF 96365 were from Tocris (Ellisville, MO).

To prepare retinal slices for electrophysiological experiments, a section of the eyecup was placed vitreal side down on a piece of filter paper (2×5 mm, AAWP, 0.8 mm pores, Millipore, Bedford, MA, USA) and isolated in cold saline solution. Slices were prepared under infrared illumination using Gen III image intensifiers (Nitemate NAVe, Litton Industries, Tempe, AZ). Retinal slices (125 µm) for both electrophysiological and imaging experiments were cut with a razor blade tissue chopper (Stoelting, Wood Dale, IL, USA) and placed in a recording chamber for viewing of the retinal layers with an upright fixed stage microscope (Olympus BHWI, Tokyo, Japan with 40X, 0.7 NA objective or Nikon E600 FN, Japan with 60X, 1.0 N.A. objective). Slices were superfused at ∼1 ml/min with an oxygenated solution containing (in mM): 111 NaCl, 2.5 KCl, 1.8 CaCl_2_, 0.5 MgCl_2_, 10 HEPES, 5 glucose, 0.1 picrotoxin, 0.001 strychnine (pH 7.8).

### [Ca^2+^]_i_ measurements

Isolated photoreceptors were loaded with 2–5 µM fura-2 AM for 10 minutes and washed for 20 minutes. This indicator has a dissociation constant (Kd) of 224 nM which is close to resting [Ca^2+^]i and provides large changes in signal for [Ca^2+^]i starting from resting levels in light-adapted photoreceptors [26]. Fluorescence signals were acquired on an inverted microscope (Nikon Ti) using a dry 40× objective (N.A. 0.8) or an oil 100× objective (N.A. 1.2) and an upright microscope (Nikon E600FN) using a 40× water (N.A. 0.8) lens. The regions of interest (ROI) were positioned onto the cell body of the cone IS, unless otherwise indicated. In a subset of experiments, data was acquired simultaneously from synaptic terminal, cell body and ellipsoid regions. Image acquisition was generally binned at 3×3 or 4×4 pixels and was run at 0.5–4 Hz by cooled CCD cameras (CoolSnap HQ2; Photometrics, Tucson, AZ). Cameras were controlled by commercial software (NIS Elements, Melville, NY). [Ca^2+^]_i_ was calculated after subtraction of the background fluorescence by measuring the ratio of the two emission intensities for excitation at 340 and 380 nm. Numerical ratio information was exported into a data analysis program (Igor Pro, Wavemetrics, Lake Oswego, OR) and used to calculate the concentration of free [Ca^2+^]_i_ in the cone cytosol.

For fura-2 quenching protocol, 50–100 µM Mn^2+^ was added to Ca^2+^-free external saline or used in 2 mM Ca^2+^-containing saline. Dye fluorescence was typically monitored at 360 nm, the isosbestic wavelength at which the signal is not affected by [Ca^2+^]i. Fluorescence emission induced by both 340 nm and 380 nm excitation wavelengths was quenched by Mn^2+^ (Fig. 3A). >95% of the de-esterified fura-2 was cytosolic, as subsequent addition of ionomycin induced a negligible additional quench (Fig. 3A). The rate of quenching was estimated from the slope of fluorescence decrease through a linear fit (y = ax+b) (Igor Pro 7.0); the result was plotted as ratio between a_1_ (slope in Mn^2+^)/a_2_ (slope in control saline) ×100. Because these non-ratiometric measurements were affected by bleaching of the indicator dye (Fig. 3B), the data was corrected by subtracting the bleaching contribution estimated by the slope difference (a_0_) from first and last exposures to control saline, quantifying the effect of tested compounds as: [(a_1_−a_0_)/(a_2_−a_0_) ×100].

Free [Ca^2+^] levels were calibrated *in vivo* with 10 µM ionomycin in 0 and 10 mM [Ca^2+^]_o_ saline using the standard relationship and a K_d_ for Ca^2+^ binding to fura-2 of 224 nM [e.g., 26]. “Low baseline [Ca^2+^]i cones are defined as having resting [Ca^2+^]i under 80 nM, typically around 50 nM. “High-baseline” cells are defined as having resting [Ca^2+^]i>100 nM. Data for cells which did not complete the calibration process is presented as 340/380 nm ratios. All pooled data is presented as mean±S.E.M. Significance was determined using the t-test (Instat 3; GraphPad, La Jolla, CA).

### Electrophysiology

Whole-cell recordings were obtained using 8–15 MΩ patch electrodes pulled from borosilicate glass (1.2 mm O.D., 0.95 mm I.D., with internal filament, World Precision Instruments, Sarasota, FL) on a PP-830 micropipette puller (Narishige USA, East Meadow, NY). The pipette solution contained (in mM): 94 Cs gluconate, 9.4 TEACl, 1.9 MgCl_2_, 9.4 MgATP, 0.5 GTP, 5 EGTA, 32.9 HEPES (pH 7.2). The osmolarity was measured with a vapor pressure osmometer (Wescor, Logan, UT) and adjusted, if necessary, to ∼242 mOsm.

Cones were voltage clamped at −70 mV and horizontal cells at −60 mV using a Multiclamp patch-clamp amplifier (Axon Instruments, Foster City, CA). Recording pipettes were positioned with Huxley-Wall micromanipulators (Sutter Instruments, Novato, CA). Currents were acquired using a Digidata 1322 interface and pClamp 9.2 software (Axon Instruments). The calcium current was recorded by subtracting the passive Cm and Rm using P/8 subtraction protocol. Cell types were distinguished by morphological and physiological criteria [69]. Charging curves for cones and many horizontal cells could be fit by single exponentials, indicating a compact electrotonic structure and suggesting that horizontal cells were largely uncoupled from their neighbors in the retinal slice preparations used for these studies.

Cone-dominated light responses were evoked using light flashes from a red LED or 680 nm light from a tungsten light source applied in the presence of a blue (480 nm) background light that strongly suppresses rod responses but only slightly diminishes cone responses [70]. Strong cone input was further indicated by the rapid response at light offset and absence of slow rod tails.

### Immunostaining

Immunostaining procedures were performed as described previously [71]. Fixed transverse sections of the retina were washed in PB for 15 min before permeabilization and blocked in 0.5% Triton X-100 and 10% goat serum. Polyclonal antibodies against STIM1 were purchased from Sigma and used at 1∶100. The monoclonal mouse SV2 antibody was developed by K. Buckley and obtained from the Developmental Studies Hybridoma Bank (Iowa City, IO). The secondary antibodies utilized were goat anti-mouse or goat anti-rabbit IgG (H+L) conjugated to fluorophores (Alexa 488 and Alexa 594 conjugates, Invitrogen), diluted 1∶500 or 1∶1000 or goat anti-mouse Cy3 from Jackson ImmunoResearch at 1∶1000. After incubation, sections on slides were washed in PBS and mounted with Vectashield (Vector, Burlingame, CA). Negative controls for non-specific staining of secondary antibodies were performed for every set of experiments by omitting the primary antibody. Immunofluorescent images were acquired at depths of 12 bits on a confocal microscope (Zeiss LSM 510) using 488 nm Ar and 594 nm He/Ne lines for fluorophore excitation, suitable band-pass or long-pass filters for emission detection and a 40×/1.2 NA oil objective.

### Statistics

All pooled data is presented as mean±S.E.M. The significance of results from experiments involving populations of cells was evaluated with the standard *t*-test. Pre- and post-drug treatment pairs for those samples were tested by the paired Student's *t* test. The significance of means of three different treatments was determined with one-way ANOVA and the Bonferrioni's posthoc and Dunnett's test. The degree of significance is indicated by asterisks: *p<0.05; **p<0.001; ***p<0.0001.

## Supporting Information

Figure S1 The L-type Ca2+ channel antagonist nifedipine inhibit HC PSCs but L-cis-diltiazem does not. A. Nifedipine (30 µM) inhibits cone ICa and PSCs recorded simultaneously in a post-synaptic HC. The cone was stimulated with a depolarizing test pulse (100 ms) from −70 to −10 mV. B. L-cis-diltiazem (10 µM) had no effect on cone ICa or HC PSCs recorded in a different cone/HC pair.
(2.70 MB TIF)

Video S1 L-type channel activation mediates polarized Ca2+ entry into the cone inner segment. Superfusion with 20 mM KCl evoked a large Ca2+ increase in the synaptic terminal, followed by [Ca2+]i elevation in the soma and the subellipsoid.
(3.98 MB AVI)

Video S2 Following store depletion, [Ca2+]i elevations are first observed in the cell body, followed by the synaptic and subellipsoid regions of the cone inner segment.
(5.69 MB AVI)
